# Aptamer-Driven Toxin Gene Delivery in U87 Model Glioblastoma Cells

**DOI:** 10.3389/fphar.2021.588306

**Published:** 2021-04-15

**Authors:** Luana di Leandro, Francesco Giansanti, Sabrina Mei, Sara Ponziani, Martina Colasante, Matteo Ardini, Francesco Angelucci, Giuseppina Pitari, Michele d’Angelo, Annamaria Cimini, Maria Serena Fabbrini, Rodolfo Ippoliti

**Affiliations:** ^1^ Department of Life, Health and Environmental Sciences, University of L’Aquila, L’Aquila, Italy; ^2^ Ministry of Instruction, University and Research, Rome, Italy

**Keywords:** glioblastoma, AS1411 aptamer, DNA-based gene delivery, saporin, nucleolin

## Abstract

A novel suicide gene therapy approach was tested in U87 MG glioblastoma multiforme cells. A 26nt G-rich double-stranded DNA aptamer (AS1411) was integrated into a vector at the 5′ of a mammalian codon-optimized saporin gene, under CMV promoter. With this plasmid termed “APTSAP”, the gene encoding ribosome-inactivating protein saporin is driven intracellularly by the glioma-specific aptamer that binds to cell surface-exposed nucleolin and efficiently kills target cells, more effectively as a polyethyleneimine (PEI)-polyplex. Cells that do not expose nucleolin at the cell surface such as 3T3 cells, used as a control, remain unaffected. Suicide gene-induced cell killing was not observed when the inactive saporin mutant SAPKQ DNA was used in the (PEI)-polyplex, indicating that saporin catalytic activity mediates the cytotoxic effect. Rather than apoptosis, cell death has features resembling autophagic or methuosis-like mechanisms. These main findings support the proof-of-concept of using PEI-polyplexed APTSAP for local delivery in rat glioblastoma models.

## Introduction

Gliomas and particularly Glioblastoma multiforme (GBM) are incurable diseases with a low life expectancy for affected patients, whose treatment is currently limited to surgery and chemotherapy (Stupp and Weber, 2005). These aggressive tumors generally undergo recurrences within 12–18 months. Actually, the first line treatment for GBM patients is temozolomide. This DNA damaging agent is particularly useful in a subclass of glioblastoma cells, those possessing a methylated promoter for the gene of O6-Methylguanine-DNA Methyltransferase (MGMT) that is involved in damage repair induced by alkylating agents. Although the therapeutic effects may be relevant to these patients, efficacy is still limited to a short time range. Furthermore, the intrinsic heterogeneity of glioblastoma cells defines an urgent need to find new targets available for an efficient multiple drug-targeting approach.

We have previously demonstrated that nucleolin, that is normally found in the cell nucleolus as a histone chaperone, can be considered a good candidate for diagnosis and treatment of gliomas (Benedetti et al., 2015; Dhez et al., 2018), being exposed at the cell surface in tumor cells (Storck et al., 2007).

In recent years, aptamers against nucleolin have been developed to potentially drive drug delivery to cancer cells.

Aptamers are small nucleic acid molecules that can specifically bind to molecular targets (from small molecules to proteins and nucleic acids), often selected from random libraries through a procedure known as SELEX (Systematic Evolution of Ligands by Exponential enrichment) (Ellington and Szostak, 1990; White et al., 2001). Aptamers that recognize cell surface receptors or proteins have been successfully used to deliver various cargoes including toxins and small interfering RNA (siRNA) (Zhu et al., 2015; Kruspe and Giangrande, 2017) or used for cancer imaging, diagnosis (Ruiz Ciancio et al., 2018) and eventually cure SARS coronavirus (SARS-CoV) (Asha et al., 2018); notably, an anti-vascular endothelial growth factor aptamer has been approved for the treatment of age-related macular degeneration in 2004 (Ng et al., 2006).

Biological effects of this type of small molecules in cancer cells include induction of cell cycle arrest, inhibition of NF-kB signaling, induction of tumor gene expression, and reduction of *bcl-2* expression (Xu et al., 2001).

AS1411 is a 26-mer G-rich DNA aptamer used as a targeting agent to deliver nanoparticles, oligonucleotides, and small molecules into cancer cells overexpressing nucleolin (Bates et al., 2009; Bates et al., 2017; Esposito et al., 2018). It binds to the cell surface nucleolin (Mongelard and Bouvet, 2010; Reyes-Reyes et al., 2010; Reyes-Reyes et al., 2015; Bates et al., 2017) leading to a selective uptake, presumably by macropinocytosis (Reyes-Reyes et al., 2010; Gao et al., 2014) and further disruption of nucleolin-mediated intracellular trafficking (Koutsioumpa and Papadimitriou, 2014). Unconjugated AS1411 has shown activity against a wide range of solid and blood cancers in preclinical experiments, and from 2005 to 2011, Aptamera then called Antisoma, a british biotechnology company, started a Phase 1 clinical trial with AS1411 (renamed ACT-GRO-777) and performed a Phase 2b trial targeting renal carcinoma and acute myeloid leukemia obtaining excellent results (Laber et al., 2007; Rizzieri et al., 2010; Rosenberg et al., 2014). Actually, however, there are no further clinical trials with ACT-GRO-777 after Phase 2b was terminated.

One of the most clear-cut results obtained in these trials has been the observed time course of AS1411 effects against cancer cells. In fact, unlike other chemotherapeutic agents, maximum cytotoxicity was reached only after long AS1411 exposures to cancer cell lines (2–4 days), therefore the patients in clinical trials were treated with a continuous infusion of AS1411 for 4–7 days with steady-state plasma concentrations in the range of 1–6 µM (Rizzieri et al., 2010).

Although “nude” AS1411 clinical trials have shown promising results, this molecule has been further explored i) for cancer-selective drug delivery, directly attaching AS1411 to gold nanostructures (Rosenberg et al., 2014; Malik et al., 2015), small chemotherapy drugs (Lale et al., 2014; Taghdisi et al., 2016; Abnous et al., 2018), peptides (Rajabnejad et al., 2018), polymers (Yang et al., 2018) and ii) used as an imaging agent by linking, for example, quantum dots (Alibolandi et al., 2014), PET isotopes (Li et al., 2014) and Raman nanoprobes (Wen et al., 2019). Graphene oxide, Doxorubicin, Gd_2_O_3_ linked to AS1411 were also used as theranostics agents (Mosafer et al., 2017; Li et al., 2018).

AS1411-linked conjugates have been tested in animal models for studying their therapeutic effects. Stable nanoparticles consisting of 6 nm gold nanospheres coated with 40 strands of AS1411 block tumor growth in nude mice bearing both human breast cancer xenografts and neuroblastoma showing an increase of anti-proliferative activity as compared to unconjugated AS1411 (Rosenberg et al., 2014). U87MG cells treatment with AS1411-functionalized poly (l-γ-glutamyl-glutamine)-paclitaxel (PGG-PTX) nanoconjugates showed a 3.5 fold greater cell growth inhibition than that of unconjugated PGG-PTX; this was due to AS1411-nucleolin mediated endocytosis of PGG-PTX, and, indeed, the AS1411 conjugation enhanced the accumulation of AS1411-PGG-PTX nanoconjugates in different organs of glioblastoma-bearing nude mice, after 24 h intravenous injection. The median survival time of mice within the AS1411-PGG-PTX nanoconjugate group was significantly longer than that of the PGG-PTX nanoconjugate mice group, showing higher numbers of apoptotic cells detected *in vivo* in the AS1411-PGG-PTX group (Luo et al., 2017).

In this paper, we describe the use of the aptamer AS1411 (APT) DNA sequence embedded in a plasmid to deliver the gene of the plant toxin saporin into glioblastoma cancer cells.

Saporin is a plant ribosome-inactivating protein (RIP) (Polito et al., 2013) used for the construction of immunotoxins (ITxs) obtained either *via* chemical conjugation of the toxic domain to monoclonal antibodies (moAbs) or by generating genetic fusions with antibody fragments able to direct the chimeric toxin against cancer cells (Giansanti et al., 2010; Lombardi et al., 2010; Errico Provenzano et al., 2016; Giansanti et al., 2018). Several studies, based on saporin-immunotoxins used to treat hematological malignancies, such as leukemias and lymphomas, demonstrated clinical efficacy and a great selective toxicity with IC_50_ values in the nano-pico molar range triggering an increase of hundred to thousand-fold in cell death, as compared to cells exposed to seed-extracted saporin alone (Polito et al., 2011; Polito et al., 2017). However, the clinical efficacy was often limited by side effects observed in patients, including antibody production against the plant toxin or Human Anti Mouse Antibody (HAMA) response to the moAb used as targeting system (MacDonald and Glover, 2005; Kreitman, 2006; Pastan et al., 2007; FitzGerald et al., 2011). To overcome the immunogenicity-related problems, several studies aimed at reducing the patient’s immune response were conducted such as with de-immunized plant RIP Bouganin (Entwistle et al., 2013) aimed also at identifying saporin critical epitopes (Giansanti et al., 2018), and to produce saporin recombinant chimeras (Lombardi et al., 2010).

A novel procedure, known as “suicide gene therapy” takes advantage from plasmids expressing cytosolic saporin (Zarovni et al., 2007) or related toxins. In a recent study, two toxin-based plasmids, pGEL (gWIZgelonin) and pSAP (gWIZ-Saporin) were tested on several cancer lines (MDA-MB-435, U87MG, 9L, HeLa) using polyethylenimine (PEI) as the transfection agent. The results showed a potent cytotoxic effect in all the tested cell lines at a gene concentration of only 2 µg/ml (Min et al., 2016). However, following transfection, the toxin present in the cytosolic compartment lead to a high level of cytotoxicity both in cancerous and non-cancerous cells, due to the non-selective mode of DNA delivery. Although the efficacy of this type of gene delivery allows for the use of very low saporin-DNA concentrations, it would be essential to develop a more selective delivery system(s) to avoid leakage of the toxin. Indeed good results have been achieved by Sama et al. (2018) using a nanoplex (peptide directed delivery) for a plasmid carrying the saporin gene that proved to be able to kill neuroblastoma cells *in vitro* and *in vivo* only when the nanoplexed DNA was given in the presence of the transfection agent sapofectosid. More recently the antitumoral activity in mice models of a plasmid carrying the saporin gene linked to Lipid-protamine DNA nanoparticles decorated with a peptide (U11), directed toward human urokinase (Salvioni et al., 2020) was demonstrated.

To further explore this possibility, here we constructed an expression cassette embedded in a standard vector [pBlueScript II KS(+)] including AS1411 at the 5′ and wild type (SAP) sequence or catalytically inactive mutant (SAPKQ) saporin gene (codon-optimized for efficient expression in mammalian cells) at the 3′, generating APTSAP/APTSAPKQ. As a control, we also produced a pBlue_SAP construct lacking the AS1411 DNA sequence. The cytotoxic effects of all these DNA constructs were tested on glioblastoma U87 cell line. The results clearly show the high efficiency of APTSAP, in inducing cell death after 96 h of treatment (IC_50_ 1.30 × 10^−8^ ± 2.42 × 10^−9^ mol/L) as compared to the toxicity of AS1411 alone (IC_50_ 4.30 × 10^−6^ ± 1.70 × 10^−6^ mol/L), while pBlue_SAP was, as expected, devoid of any toxicity. Cell death may be also induced by non-apoptotic mechanisms. Toxicity is specifically directed against cells expressing cell surface nucleolin, since nucleolin-negative cells are unaffected. Furthermore, APTSAPKQ, the catalytically inactive form of APTSAP, does not show any cell toxicity, as expected (Fabbrini et al., 2017).

Moreover, we demonstrated here that upon cell entry, APTSAP effectively induces the synthesis of the toxin saporin, thus leading to cell intoxication. We further show that APTSAP enters target cells presumably by forming a G-quadruplex structure, as revealed by microscopy in the presence of proto-porphyrin IX, confirming that this structure can act as a delivery guide for saporin-DNA to reach the surface of U87 cells where it can be recognized by nucleolin. A polyplexed complex of PEI/APTSAP plasmid gave an efficient cytotoxicity in U87 cells after just 24 h treatment, as compared to 96 h with a naked APTSAP DNA, supporting the possibility that a potential treatment of model GBM *in vivo* might be explored either by systemic treatment (GBM shows blood-brain barrier alterations) or in alternative by a local treatment. Thus we believe that this aptamer-mediated toxin gene delivery, a protein-free approach which causes tumor cell death, may represent an innovative tool to fight GBM.

## Materials and Methods

### Biochemicals

Cell Death Detection Elisa kit was purchased from Roche (Penzberg, Germany). Sodium dodecylsulphate (SDS), Tween-20, Polyethylenimine branched (PEI), Fluorescein-5-isothiocyanate (FITC)-labelled anti-mouse IgG antibodies, PVDF membranes, primary anti-p62, anti-LC3I/II, anti-Actin antibodies, Dulbecco’s Modified Eagle’s Medium (DMEM) were purchased from Sigma (St. Louis, Mo, United States). Maxi-prep plasmid extraction kit was purchased from QIAGEN (Hilden, Germany). Chemioluminescence (ECL) detection kit was purchased from Amersham Biosciences (Amersham/GE Healthcare, Italy). Fetal Bovine Serum (FBS), Trypsin-EDTA solution, streptomycin-penicillin, L-Glutamine were purchased from Euroclone SPA (Pero, MI, Italy). Micro BCA protein detection kit was from Pierce (Rockford, IL, United States). CellTiter 96AQueous One Solution Cell proliferation Assay was purchased from Promega (Madison, United States). Sodium chloride was purchased from Montplet and Esteban SA (Barcelona, Espana).

Protoporphyrin IX disodium salt was from Sigma Aldrich.

TRIsure™ was from BIOline (Meridian Bioscience, United States). All the reagents used for RT-PCR experiments were purchased from ThermoFisher Scientific (United States).

### DNA Constructs

**FIGURE 1:**
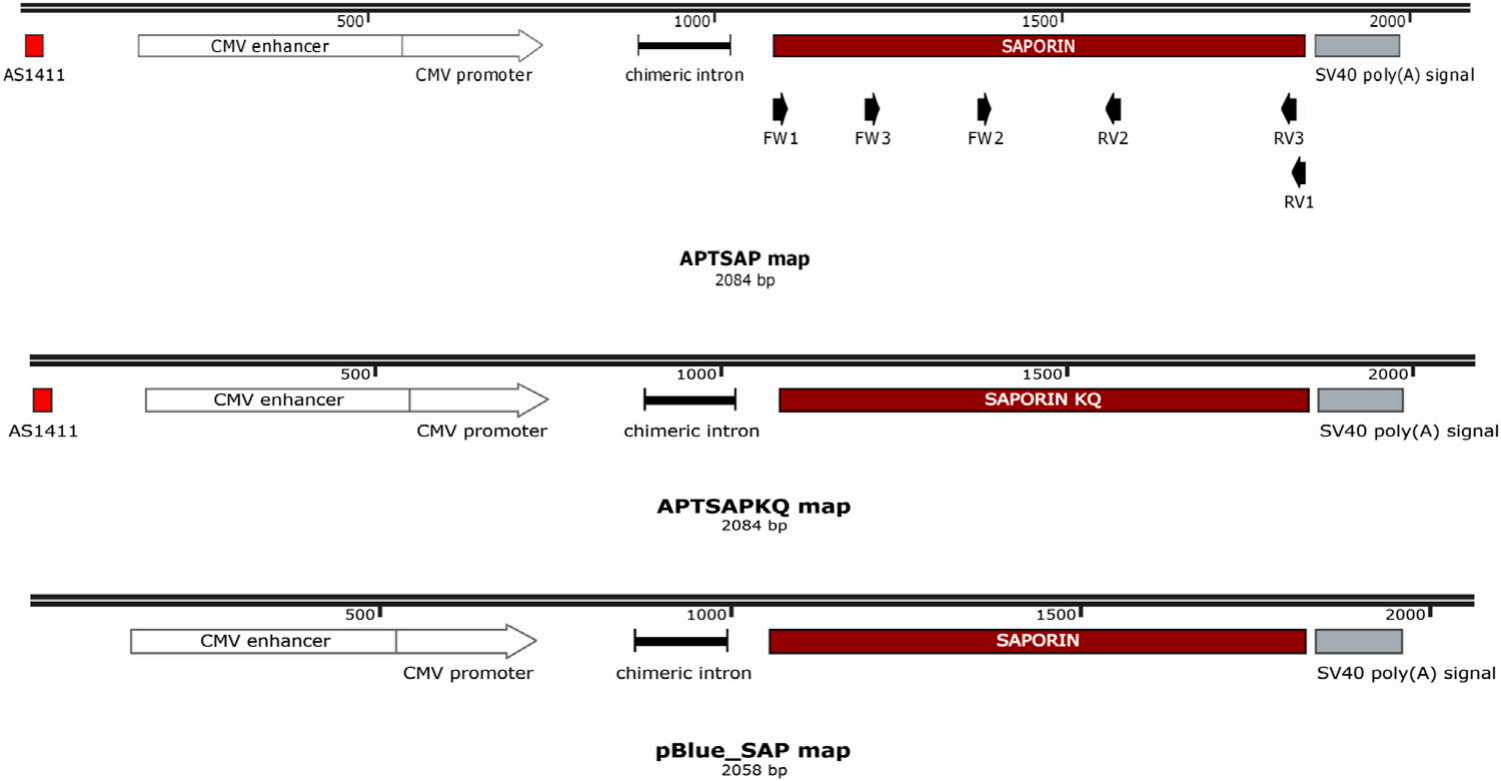
Schematic representation of APTSAP, APTSAPKQ and pBlue_SAP DNA sequences. The nucleotide sequence of AS1411 [5′-d (GGT​GGT​GGT​GGT​TGT​GGT​GGT​GGT​GG)-3] was placed at the 5′ terminus of a synthetic construct containing the CMV promoter, the saporin gene, a chimeric intron derived from pCI vector, and a poly-adenylation signal at the 3′ terminus and inserted into pBluescript II KS (+) vector using a SmaI restriction site. In the APTSAPKQ construct the saporin gene sequence was substituted with the saporin KQ mutated sequence. The pBlue_SAP construct lacks the 5′ AS1411 aptamer sequence. In the scheme we also indicated the PCR primers at their relative positions (FW1/RV1, FW2/RV2, FW3/RV3). This map was created by the SnapGene Viewer freeware software (“SnapGene software” from Insightful Science; available at http://snapgene.com).

APTSAP, APTSAPKQ and pBlue_SAP vectors DNA synthesis and codon optimization for mammalian cell expression were from GenScript (United States), while the single strands corresponding to AS1411 (ssAPT) was purchased from Eurofins Genomics (Germany). In APTSAP and APTSAPKQ the nucleotide sequence of AS1411 (5′-d (GGT​GGT​GGT​GGT​TGT​GGT​GGT​GGT​GG)-3) was placed at the 5′ termini of a synthetic construct containing the CMV promoter, saporin optimized gene(s), a chimeric intron derived from pCI vector, and a poly-adenylation signal at the 3′, subcloned into pBluescript II KS (+) plasmid. pBlue_SAP represents the same construct as in APTSAP except for the absence of the AS1411 targeting sequence. A scheme of the APTSAP, APTSAPKQ and pBlue_SAP DNA constructs is reported in Figure 1.

DNA plasmids were amplified in *E. coli* DH5α cells and purified by QIAGEN Maxi kit (QIAGEN, United States), denatured at 95°C for 2 min at pH 7.5 with 2.5 mM KCl, and slowly cooled at room temperature immediately before the use. The pBluescript II KS(+) nude plasmid was used as a control plasmid in all the viability experiments.

All the constructs were utilized as undigested plasmids. AS1411 was used either as a single strand (ssAPT) DNA or reconstituted with the complementary strand (dsAPT). For the annealing of ssAPT and its complementary strand, we have followed the Sigma Aldrich’s protocol: the forward ssAPT oligo (100 nmol/ml) and the reverse complementary APT oligo (100 nmol/ml) were mixed together with the annealing buffer 10 X (100 mM Tris/HCl pH 7.5, 1 M NaCl, 10 mM EDTA) and nuclease free water to appropriate volume. The oligo solution was heated to a temperature 10°C higher than the calculated melting temperature for 10 min. The annealed oligos solution (dsAPT) was removed from the heating block and was cooled slowly (approximately 1 h) to room temperature on the bench. The ssAPT and the annealed dsAPT were then denatured at 95°C for 2 min in the presence of 2.5 mM KCl, and slowly cooled at room temperature, immediately before use. Double stranded and G quadruplex content of ssAPT and dsAPT constructs were analyzed by visualization on 1.2% agarose gel (Supplementary Figure S1) in the presence of ethidium bromide. Furthermore the formation of G quadruplex in ssAPT and dsAPT was studied by fluorescence emission of DNA-protoporphyrin IX complexes (Li et al., 2010) and spectra recorded on Nanodrop 3300 fluorimeter (ThermoFisher Scientific, United States) following the increase of fluorescence at 632 nm, or by absorption spectra (Li et al., 2010) recorded on a Cary 60 spectrophotometer (Agilent Technologies, United States), following the increase of absorption in the Soret region at 415 nm (Supplementary Figure S1).

### Cell Cultures

Cultures of U87MG cells (ATCC HTB-14 glioblastoma multiforme cell lines) and 3T3 cells (ATCC CRL 1658) were grown in Dulbecco’s Modified Eagle’s Medium (D-MEM) supplemented with 10% heat-inactivated fetal bovine serum (FBS), 0.1 mg/ml penicillin/streptomycin and 2 mM l-Glutamine. The cell cultures were incubated at 37°C in a 5% CO_2_ humidified atmosphere. All experiments were carried out seeding the cells on day 0 at 1600 cells/cm^2^. Cells were treated with ssAPT, APTSAP, and APTSAPKQ vector constructs carrying the gene of the saporin toxin added in complete culture medium for 96 h.

### Cytotoxicity Assays

U87MG and 3T3 cells were grown to 70% confluency in 96-well culture plates. Before starting the experiment, the cells were washed twice with PBS and then incubated in 200 μl complete DMEM high glucose medium (Euroclone S. p.A., Italy) containing from 0 to 0.1 μmol/L (0, 0.001, 0.010, 0.020, 0.050, 0.1 μmol/L) APTSAP, APTSAKQ, pBlue_SAP and control pBlueScript II KS(+) and from 0 to 50 μmol/L (0, 0.001, 0.010, 1, 10, 50 μmol/L) ssAPT, for 96°h. In some experiments dsAPT was tested in the same concentration range as ssAPT. The viability of cells was then examined using a modified 3-(4,5-dimethylthiazol-2-yl)-2,5-diphenyl tetrazolium bromide assay (CellTiter 96AQueous One Solution Reagent, Promega, Madison WI). The color change was measured at 490 nm after 1 h of incubation in a spectrophotometric microplate reader (Infinite F200 Tecan). 3T3 cells have been used as controls (nucleolin negative cells) according to the literature (Feng et al., 2011; Yin et al., 2012; Wu et al., 2013; Charoenphol and Bermudez, 2014; Hong et al., 2018) in comparison with U87 cells.

### Apoptosis Measurements

Determination of cytoplasmic histone-associated DNA fragments was performed by using the Cell Death Detection ELISA Kit, following the manufacturer’s instructions and using mouse monoclonal antibodies directed against DNA and histones, respectively. The absorbance peak at 405 nm was measured in a spectrophotometric microplate reader (Infinite F200 Tecan).

### Protein Assay

The total protein content of cell cultures was assessed using the bicinchoninic acid method (BCAProtein Reagent, Sigma), and the determination of absorbance at 570 nm was assessed using a spectrophotometric microplate reader (Infinite F200 Tecan). The amount of protein was measured by extrapolation from a standard curve using BSA as standard.

### Western Blotting

Lysates from control and treated sub-confluent cells were run on 12% polyacrylamide SDS PAGE denaturing gels. Protein bands were transferred onto polyvinylidene difluoride (PVDF) sheets by electrophoretic transfer. Non-specific binding sites were blocked with a blocking solution: 5% (w/v) non-fat dry milk in Tris-buffer saline (TBS: 20 mM Tris–HCl, pH 7.4, containing 150 mM NaCl) containing 0.05% (v/v) Tween-20 (TBS-T) for 1 h at RT. Membranes were then incubated overnight at 4°C with the following primary antibodies, diluted with TBS containing 0.1% Tween 20 (TBS-T) and 5% non-fat dry milk: anti-p62/SQSTM1 polyclonal antibody (1:1000, Sigma Aldrich), anti-LC3-I/II polyclonal antibody (1:1000, Sigma Aldrich) and anti-Actin polyclonal antibody (1:1000, Sigma Aldrich).

Membranes were then extensively washed with TBS-T and incubated with goat anti-rabbit peroxidase-conjugated secondary antibody (1:2000, in blocking solution, Santa Cruz) for 2 h. After rinsing with TBS-T, the specific immune complexes were detected by enhanced chemiluminescence (ECL) (Amersham Biosciences). Then, blot analyses were performed using ImageJ software (NIH, Bethesda, United States; http://rsb.info.nih.gov/ij/index.html).

### Immunofluorescence and Confocal Microscopy Analyses

Cells grown on coverslips were fixed in 4% paraformaldehyde in PBS for 10 min.

Non-specific binding sites were blocked with PBS containing 3% BSA for 30 min at RT, followed by incubation with primary antibodies (rabbit) anti-gp 273 (1:100) 1 h at R.T. After extensive washings with PBS, cells were treated with fluorescein-labeled anti-rabbit IgG secondary antibodies (1:1000 in PBS containing 3%, w/v, BSA) for 30 min. Nuclei were stained with Hoechst.

Coverslips were mounted with a Vectashield mounting medium and examined with a Zeiss Axio Imager A2 fluorescence microscope. For cell imaging of APTSAP-protoporphyrin IX (APTSAP-PPIX), the complex of APTSAP-PPIX was prepared as described: APTSAP plasmid was purified and resuspended in 20 mM Tris-HCl buffer pH 7.4 containing 2.5 mM KCl, heated at 95°C for 2 min, and gradually cooled to room temperature immediately before use. 5 µM of protophorphyrin IX was added in the plasmid solution at 1:1 ratio and incubated for 2 h. The APTSAP-PPIX complex was then placed over cells for 1 h. The cells were washed with PBS (Phosphate buffer saline), fixed and examined with a Zeiss Axio Imager A2 fluorescence microscope (Zeiss, Germany) and with a Leica TCS SP5 confocal microscope (Leica Microsystems, Mannheim, Germany).

### Transfection Conditions Optimization

U87MG and 3T3 cells were seeded at 1600 cells/well in 96-well culture plates and incubated in 200 μl of complete DMEM high glucose medium (10% FBS, 100 I.U./ml penicillin, 100 (μg/ml) streptomycin. and 2 mM L-glutamine) containing from 0 to 100 μg/ml of PEI (0, 10, 20, 30, 40, 50, 60, 70, 80, 90 and 100 μg/ml). Cells were exposed to the different PEI concentrations from 24 to 96 h and to a different gene to PEI ratios (1:1, 1:5 and 1:10). The viability of cells was examined using a modified 3-(4,5-dimethylthiazol-2-yl)-2,5-diphenyl tetrazolium bromide assay (CellTiter 96AQueous One Solution Reagent, Promega, Madison WI). The color change was measured at 490 nm after 1 h of incubation in a spectrophotometric microplate reader (Infinite F200 Tecan).

### Reverse Transcription Polymerase Chain Reaction

For the RT-PCR experiments, both cell lines were treated for 96 h with 0 and 20 nM of APTSAP and then total RNA was extracted from the cell pellet (3 × 10^6^ cells) by TRIsure (Bioline). Extracted RNA was subjected to a DNAse pre-treatment to remove DNA contaminants and, after that, was re-purified by the chloroform and 2-propanol method (1v CHCl_3_; 1v 2-propanol; 2,5v ethanol).

For the first strand synthesis we used the Superscript II Reverse Transcriptase from Invitrogen (Thermofisher Scientific, United States). A 20 µl reaction volume was used for 1 µg of total RNA with the following protocol: in a nuclease-free tube were added 1 µl of Oligo dT_12–18_ (500 μg/ml), 1 µg of total RNA, 1 μl 10 mM dNTP Mix (10 mM each) and water. The mixture was heated for 7 min at 65°C and then were added 4 μl of 5XFirts strand buffer, 2 µl of 0.1 M DTT, and 1 μl of RNase OUT. The mixture was incubated at 42°C for 5 min and then was added 1 µl of Superscript TM ^II^ RT. The reverse reaction was performed for 50 min at 42°C and inactivated by heating at 70°C for 15 min. For removing RNA complementary to the cDNA, 1 μl of RNase H (Thermofisher Scientific) was added to the reaction and incubated at 37°C for 20 min. To perform the PCR amplification of the saporin gene, were used the following primers couples: Fw1 5′ATG​GTC​ACC​TCA​ATA​ACC​CTT 3′ (Tm 55°C), Rev1 5′ TTA​TTT​GGG​TTT​GCC​GAG​AT 3′ (Tm 55°C), Fw2 5′AAC​TGA​CCG​CTC​TGT​TTC​C3′ (Tm 57′C), Rev2 5′ATC​TTG​CTT​CGT​TCT​TGA​CC 3′ (Tm 56°C), Fw3 5′CCA CCG TCC AAA GAG AAG TTC C 3′ (Tm 62°C) and Rev3 5′GCC GAG ATA CAT AAG CAA GCC C 3′ (Tm 62.1°C). pET11d plasmid containing the saporin gene sequence was used as a positive control. The position of each primer is reported in Figure 1.

### Statistical Analysis

Statistical analysis was performed by ANOVA test using GraphPad Prism 8 (San Diego, CA, United States). Data are expressed as mean ± S.E.M. of three different experiments.

For the IC_50_ determination we used a [inhibitor] vs. normalized response—Variable slope least squares fit. IC_50_ values were obtained from the mean of the IC_50_ of each independent experiment and reported as mean ± S.E.M.

*p < 0.033 **p < 0.0021 ***p < 0.0002 ****p < 0.0001.

## Results

### Effects of Saporin and SaporinKQ Genes Linked to AS1411 (APTSAP and APTSAPKQ) on Glioblastoma Cell Viability

The APTSAP and APTSAPKQ plasmids were prepared by designing a DNA sequence containing the mature SAP-3 gene (Zarovni et al., 2007), and the inactive saporin SAPKQ-mutant gene, under the CMV promoter control and a transcription terminator placed downstream the polyadenylation signal for improving transcription efficiency (Figure 1). The SAPKQ mutant is devoid of the N-glycosidase activity (Zarovni et al., 2007) and is characterized by a significant reduced RIP activity, due to the substitution of two active key residues, Glu176 and Arg179 with Lys and Gln, respectively (Lombardi et al., 2010). These two sequences were inserted in the pBluescript II KS(+) plasmid backbone and used to transform *E. coli* DH5α cells as a source of the recombinant plasmid to be used throughout all the experiments.

Preliminary MTS analysis of cell viability (data not shown) have been performed after cell exposure to APTSAP and APTSAPKQ at concentrations ranging from 0 to 100 nM for 24, 48, 72 and 96 h and compared the viability after exposure to AS1411 alone (ssAPT, from 0 to 50 µM). Based on these preliminary results, we then tested the effects of the various DNA concentrations after 96 h incubation.

**FIGURE 2:**
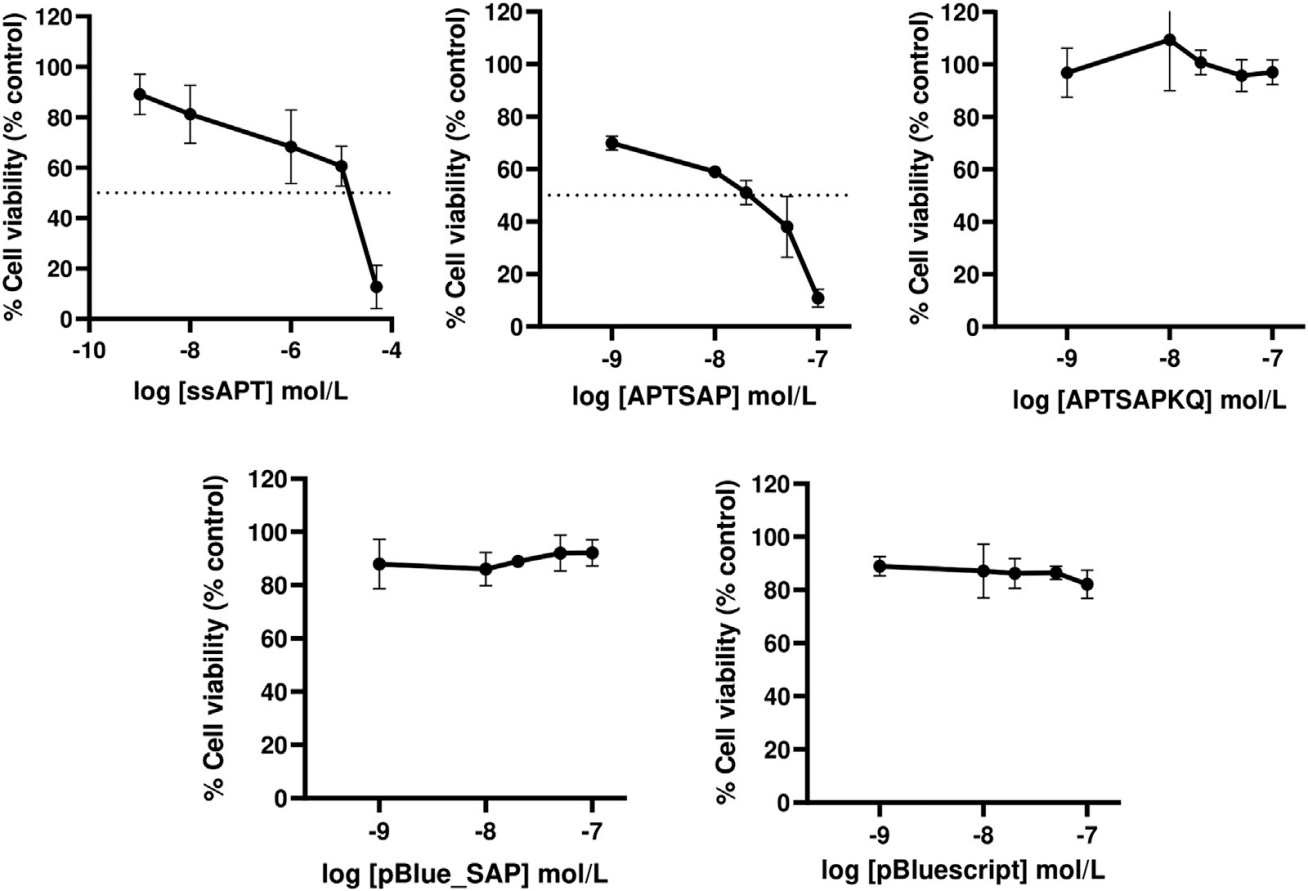
Dose-response curves showing respectively the effects of ssAPT, APTSAP, APTSAPKQ, pBlue_SAP and pBluescript II KS (+) on U87MG cells. Cells (1.6 × 10^3^/well) were seeded in 96-well plates in a final volume of 200 µl of medium (5% FBS) containing the appropriate concentrations (0, 0.001, 0.01, 1, 10, 50 μmol/L) of ssAPT or the appropriate concentrations (0, 0.001, 0.01, 0.02, 0.05 and 0.1 μmol/L) of APTSAP, APTSAPKQ, pBlue_SAP and pBluescript II KS (+) for 96 h. All the plasmids were denatured at 95°C for 2 min in 10 mM Tris/HCl buffer pH 7.5 containing 100 mM NaCl and 2.5 mM KCl immediately before use. Cell viability was examined using a MTS assay and expressed as percentage of control untreated cells. ssAPT IC_50_: 4.30 × 10^−6^ ± 1.70 × 10^−6^ M (mean ± S.E.M.); APTSAP IC_50_: 1.30 × 10^−8^ ± 2.42×10^−9^ M (mean ± S.E.M.). The data reported are representative of a set of at least three independent experiments and are reported as mean ± SD.

The results indicate a toxic activity of APTSAP in the low concentration range (1–100 nM) in contrast with a faint ssAPT effect on cell viability. As shown in Figure 2 APTSAP shows an IC50 of 1.30 × 10^−8^ M about two orders of magnitude lower than that of ssAPT alone (IC_50_ 4.30 × 10^−6^ M).

No cytotoxic effect was observed when U87MG cells were treated with the nude pBluescript or with pBlue_SAP plasmids at the same concentrations of APTSAP or APTSAPKQ, as expected (Figure 2).

Since the AS1411 aptamer sequence is in the form of double-stranded DNA in APTSAP, we also compared ssAPT (AS1411 as single-stranded DNA) with a mixture of ssAPT and its complementary strand (dsAPT, double stranded DNA) on cell viability. Both ssAPT and dsAPT proved to be active to the same extent (Supplementary Figure S2). dsAPT in the experimental conditions used produces about 95% ssAPT (Supplementary Figure S1) and it is able to adopt the G quadruplex structure, as well as ssAPT, under denaturing and annealing in the presence of K^+^ ions (Supplementary Figure S1).

**FIGURE 3:**
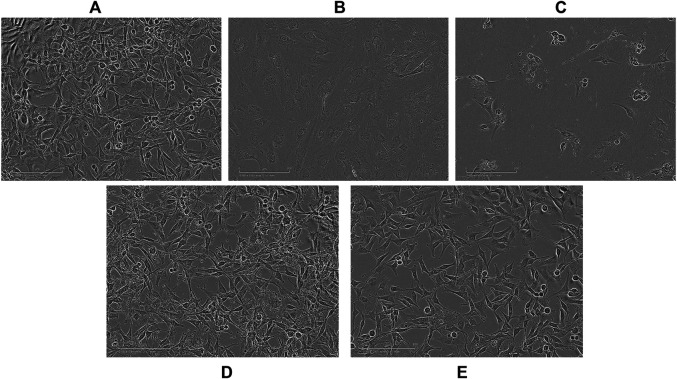
Representative images observed with an optical microscope of U87 cells after 96h of treatment. Controls **(A)**; ssAPT 25 µM **(B)**; APTSAP 20 nM **(C)**; APTSAPKQ 20 nM **(D)**; pBlue_SAP 20 nM **(E)**.

Optical images of U87 cells after 96 h exposure to APTSAP show shrinkage, cell rounding, and fragmentation, but no evidence of *apoptotic* bodies and rapid phagocytosis by neighboring cells; minor signs of morphological changes were present in cells treated with ssAPT (Figure 3). APTSAPKQ did not induce any morphological change, as well as pBlue_SAP lacking AS1411 sequence, confirming what was observed in the cytotoxicity experiments.

**FIGURE 4:**
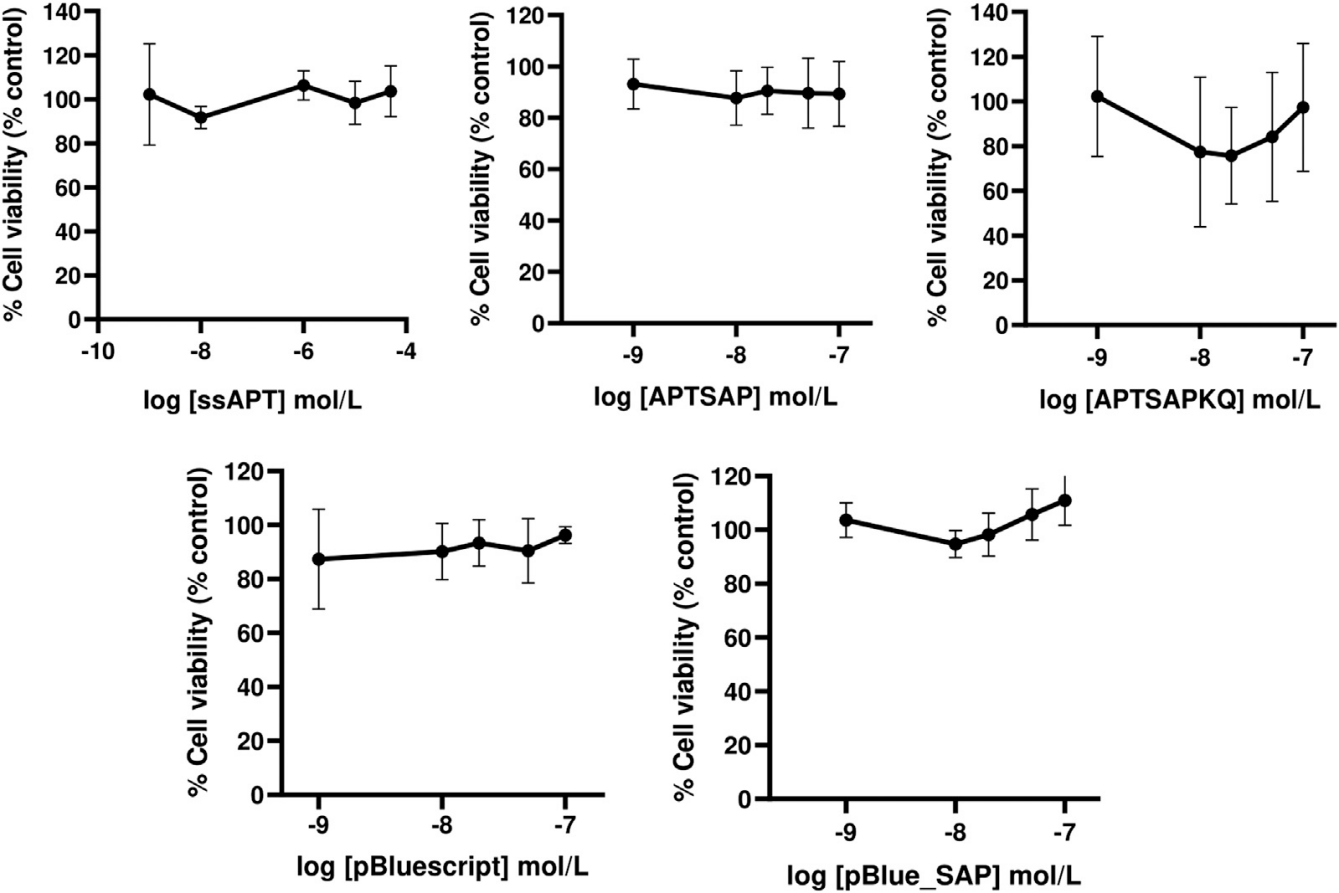
Dose-response curves showing respectively the effect of ssAPT, APTSAP, APTSAPKQ, pBlue_SAP and pBluescript II KS (+) on control NIH3T3 cells. Cells (1.6 × 10^3^/well) were seeded in 96-well plates in a final volume of 200 ul of medium (5% FBS) containing appropriate concentrations (0, 0.001, 0.01, 1, 10, 50 μmol/L) of ssAPT and appropriate concentrations (0, 0.001, 0.01, 0.02, 0.05 and 0.1 μmol/L) of APTSAP, APTSAPKQ, pBlue_SAP and pBluescript II KS (+) and for 96 h. All the plasmids were denatured at 95°C for 2 min in 10 mM Tris/HCl buffer pH 7.5 containing 100 mM NaCl and 2.5 mM KCl immediately before use. Cell viability was examined using a MTS assay and expressed as percentage of control untreated cell. The data presented are representative of a set of at least three independent experiments and are reported as mean ± SD.

**FIGURE 5:**
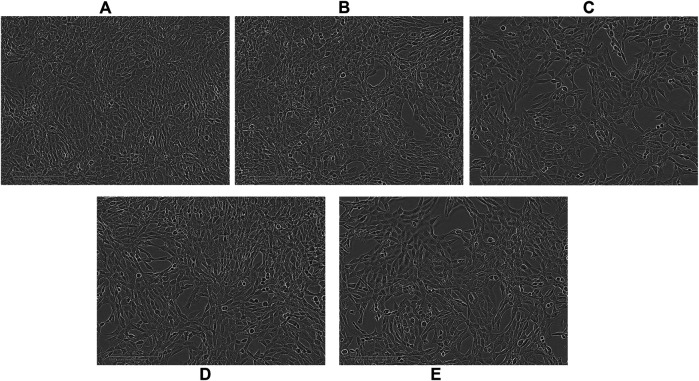
Representative images observed with an optical microscope of NIH3T3 cells after 96 h of treatment. Controls **(A)**; ssAPT 25 µM **(B)**; APTSAP 20 nM **(C)**; APTSAPKQ 20 nM **(D)**; pBlue_SAP 20 nM **(E)**.

The specificity of recognition towards cancer cells was tested by applying the same DNA constructs on 3T3 cells (Figure 4). On this cell line, we didn’t get any cytotoxicity, neither using APTSAP nor ssAPT, as well as, using the naked pBlueScript II KS(+) or pBlue_SAP plasmids. Optical microscopic images on 3T3 cells (Figure 5) confirm what was observed in the cytotoxicity experiments.

### APTSAP and APTSAPKQ Transfection Experiments

To verify whether the cytotoxicity we observed can be attributed to the specific internalization of APTSAP and consequent expression of the toxin gene, we have also carried out experiments using both APTSAP and APTSAPKQ plasmids to transfect U87 and 3T3 cells, as control, complexed with polyethyleneimine as transfection agent for up to 6 days of exposure (Nakamura, 2013).

The transfection conditions were established after viability studies of cells exposed to different PEI concentrations, from 0 to 30 μg/ml and for 0–6 days (see *Materials and Methods*). U87 and 3T3 cells were both extremely susceptible to PEI, therefore we established a PEI concentration of 10 μg/ml and a gene to PEI ratio of 1:1.

To examine whether this cationic/lipid gene delivery is selective for target cells, we have transfected in parallel NIH3T3 cells with APTSAP and ssAPT in the 1:1 ratio (gene to PEI) at the same concentration of 10 μg/ml used for U87 cells. The APTSAP and ssAPT corresponding concentrations were 8 nM and 1.2 µM, respectively.

**FIGURE 6:**
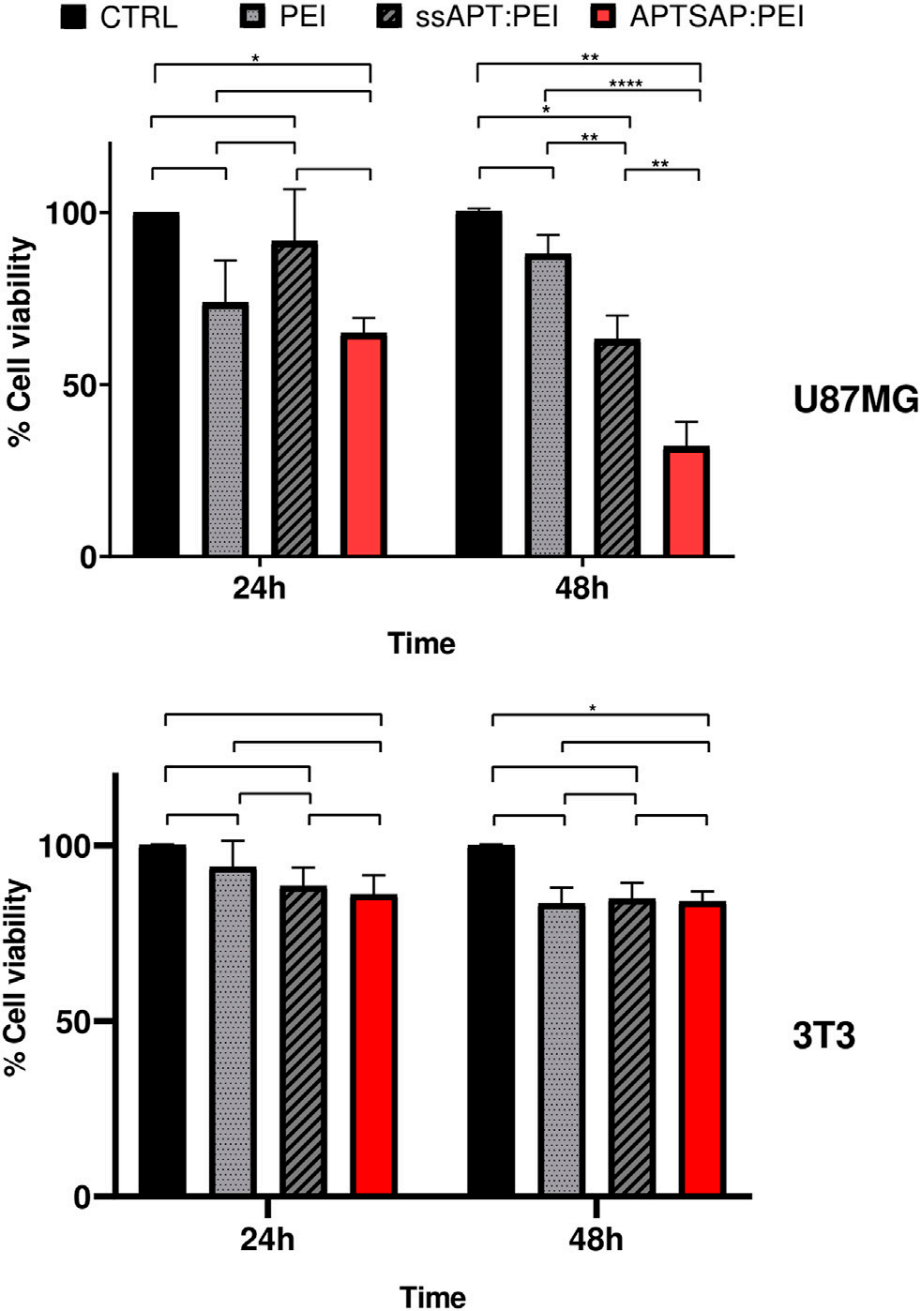
U87MG and 3T3 cell viability measurements after 24 and 48 h transfection experiments using PEI alone, ssAPT and APTSAP complexed with PEI. The gene to PEI ratio was 1:1. ssAPT concentration was of 1.2 µM; the APTSAP concentration was of 8 nM. Statistical analysis was performed by the two way ANOVA test. *p < 0.033 **p < 0.0021 ****p < 0.0001.

After 24 and 48 h treatments, cell viability was measured by the MTS colorimetric assay (Promega, United States) for assessing cell metabolic activity and compared to that obtained for U87 cells. The results are reported in Figure 6. A significant reduction in cell viability was observed for APTSAP complexes in U87 cells early at 24 h, then significantly decreasing after 48 h. In control NIH3T3 cells (Figure 6), we didn’t observe any significant difference in cell viability for all the conditions tested, except for the samples treated with APTSAP:PEI at 48 h, only if compared to the control cells.

### Cell Death Analysis

Experimental evidence demonstrated that the cell death pathways induced by saporin are, in general, due to the induction of multiple apoptotic pathways, which could be caused by different cell injuries, such as protein synthesis inhibition or DNA damage, or could be the result of oxidative stress, leading to the execution either of apoptotic or different cell death programs (i.e., autophagy or necroptosis) (Zarovni et al., 2007). Overall our results indicate that APTSAP complexes do not induce apoptosis in U87 cells *in vitro*.

**FIGURE 7:**
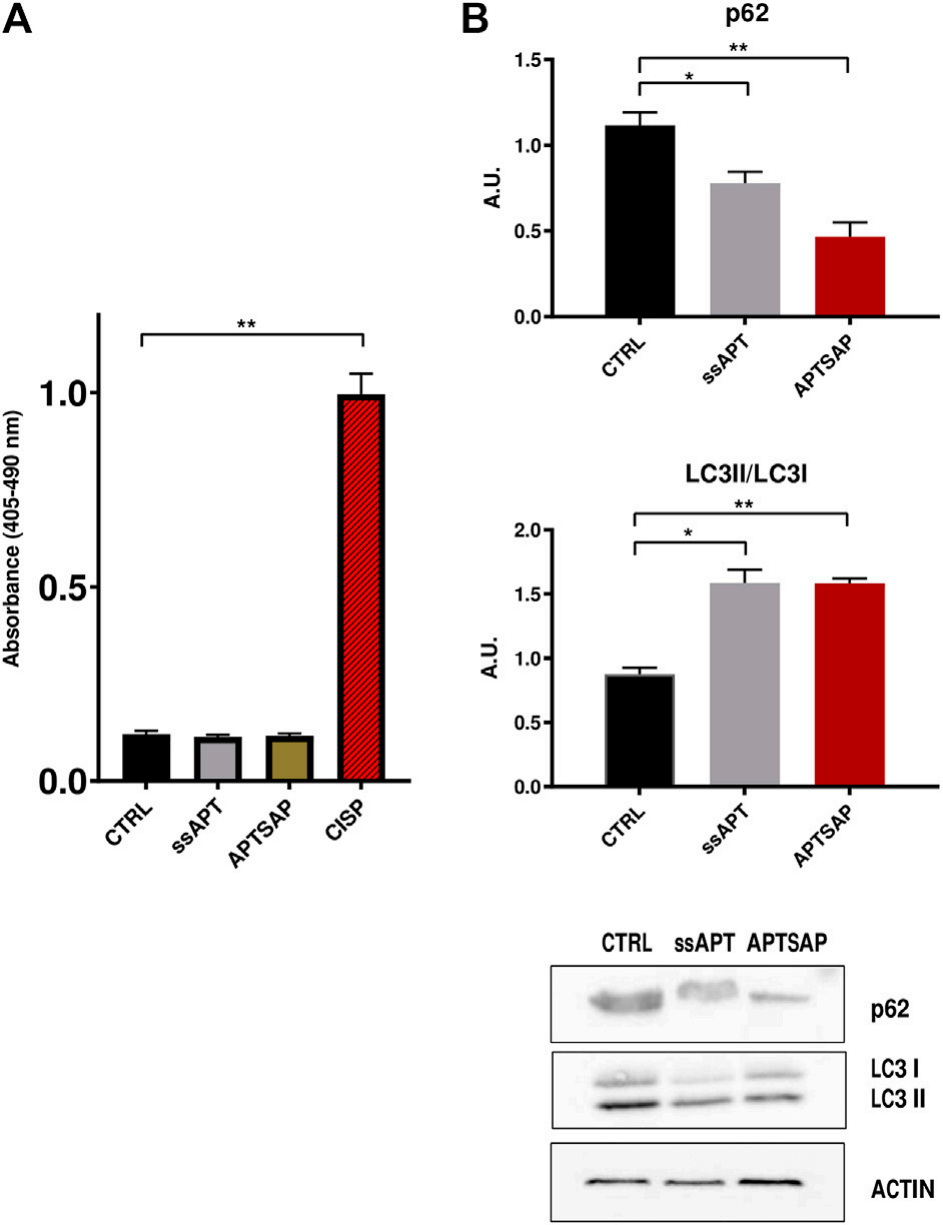
**(A)** Detection of nucleosome fragmentation in cytoplasmic fractions. Apoptosis was detected using the Cell Death detection ELISA kit. U87MG cells were treated for 96 h with APTSAP and ssAPT complexes. Cisplatin (CISP) treatment of the cells was used as a positive control. **(B)** p62 and LC3 immunoblotting analysis of U87MG cells after 96 h treatment with ssAPT (25 µM) and APTSAP (20 nM) and relative densitometric analysis. Statistical analysis was performed by the ANOVA test. *p < 0.033 **p < 0.0021.

Induction of apoptosis was measured after 96 h exposure of U87 cells to ssAPT and APTSAP complexes, by measuring the quantification of histone-complexed DNA fragments in the cytosol of treated cells. U87MG cells did not appear to be affected by the treatment (Figure 7A).

Controls show, as expected, that cisplatin (CISP) induces apoptosis in U87 cells. However, there was no significant increase in the percentage of treated cells undergoing apoptosis with respect to control cells, suggesting that the observed impairment in cell growth could also be due to other events, rather than apoptotic mechanisms in U87MG cells. To verify whether cell growth inhibition in U87MG cells could be due to cell death mechanism other than apoptosis, we then evaluated the microtubule associated protein 1A/1B light chain 3 (LC3) expression, following treatment with ssAPT or APTSAP, which is a reliable method for monitoring autophagy and autophagy-related processes (Tanida et al., 2004).

At the same time, another well recognized marker for detection of autophagy is the decrease in p62 levels (Bjørkøy et al., 2009). Both these markers were analyzed (Figure 7B, Supplementary Figures S3–S5).

The analysis of LC3II/LC3I levels in U87 cells shows an increase in their ratio in the treated cells (ssAPT and APTSAP) as compared to control cells, suggesting the presence of an autophagic activity after 96 h of treatment. Furthermore, after the same treatments, U87 cells showed a significant decrease in p62 (Figure 7B).

Recent evidence indicates that AS1411 is taken up by cancer cells *via* macropinocytosis in a nucleolin-dependent manner (Reyes-Reyes et al., 2010), stimulating the formation of large membrane vesicles, called macropinosomes, that encapsulate a large volume of extracellular fluid.

Continuous expansion of these large vacuoles, finally causes cell rupture. This process was termed “methuosis”, a particular form of cell death characterized by persistent extracellular fluid uptake by macropinosomes (Cai et al., 2013).

**FIGURE 8:**
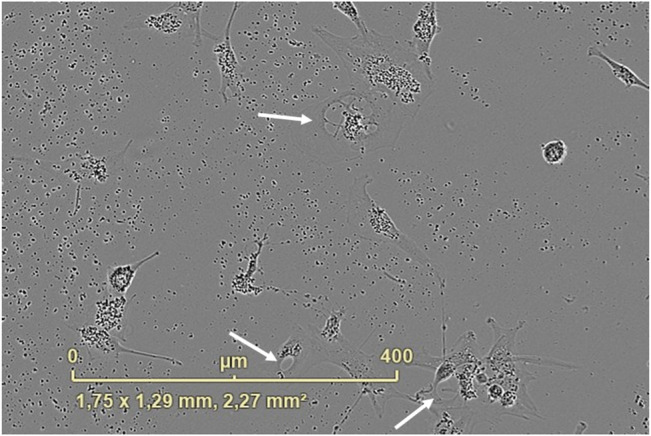
Cytoplasm vacuolization was observed after 24 h from the APTSAP transfection of U87 cells with PEI (1:1, gene to PEI ratio, 10 μg/ml). The image was recorded using the IncuCyte ZOOM^®^ live cell imaging system.

Cytoplasm vacuolization was evident when U87 cells were transfected with APTSAP/PEI. As shown in Figure 8, sizeable translucent vacuoles appeared in the cytoplasm after 24 h from transfection, leading to cell enlargement and rupture; at 48 h from transfection, an extreme cellular fragmentation is observed with a lot of cellular debris and cell detachment.

Hence, the observed cell death seems to be due to a non-apoptotic mechanism, autophagic or methuosis-like, and further experiments have to be performed to confirm the hypothesis.

### Imaging of U87MG Cells by APTSAP-Protoporphyrin IX

**FIGURE 9:**
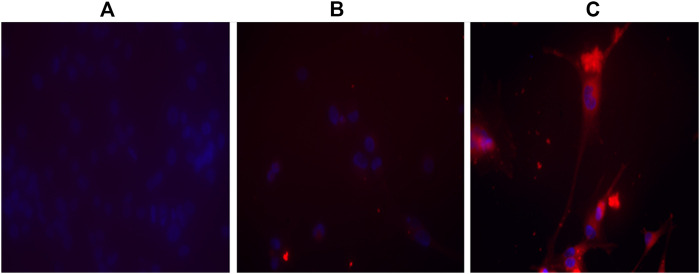
Imaging of U87MG cells by APTSAP-protoporphyrin IX. U87MG cells were incubated for 2 h with 5 µM APTSAP-PPIX complexes and 5 µM PPIX (see *Materials and Methods*) and observed under a fluorescence microscope. The strong fluorescence signal observed after APTSAP treatment was indicative of PPIX binding to G-quadruplex AS1411 conformation inside the plasmid vector. **(A)** control; **(B)** PPIX; **(C)** APTSAP-PPIX.

To verify the binding of APTSAP vectors to U87 cells, the APTSAP-protoporphyrin IX (APTSAP-PPIX) complex was used for imaging the formation of a G4-structure over the membrane of U87 cells under fluorescence and confocal microscopy. J. Ai *et al.* proposed the use of AS1411 and the fluorescent ligand PPIX to discriminate cancer cells from healthy cells and for imaging, as shown by the fluorescence enhancement of HeLa cells after the formation of APTSAP-PPIX-nucleolin complexes (Ai et al., 2012). PPIX is a G-quadruplex specific ligand able to bind AS1411 quadruplexes resulting in fluorescence enhancement (Li et al., 2010). After U87 treatment with 5 µM APTSAP-PPIX complexes, we have observed a stronger fluorescence intensity in the latter treated cells, as compared to non-treated or PPIX-treated cells (Figure 9).

The fluorescence signal indicates that i) PPIX can presumably recognize and bind the G-quadruplex structure of AS1411 inside the APTSAP vector and ii) AS1411 mediates vector entry by nucleolin trafficking into the cell.

**FIGURE 10:**
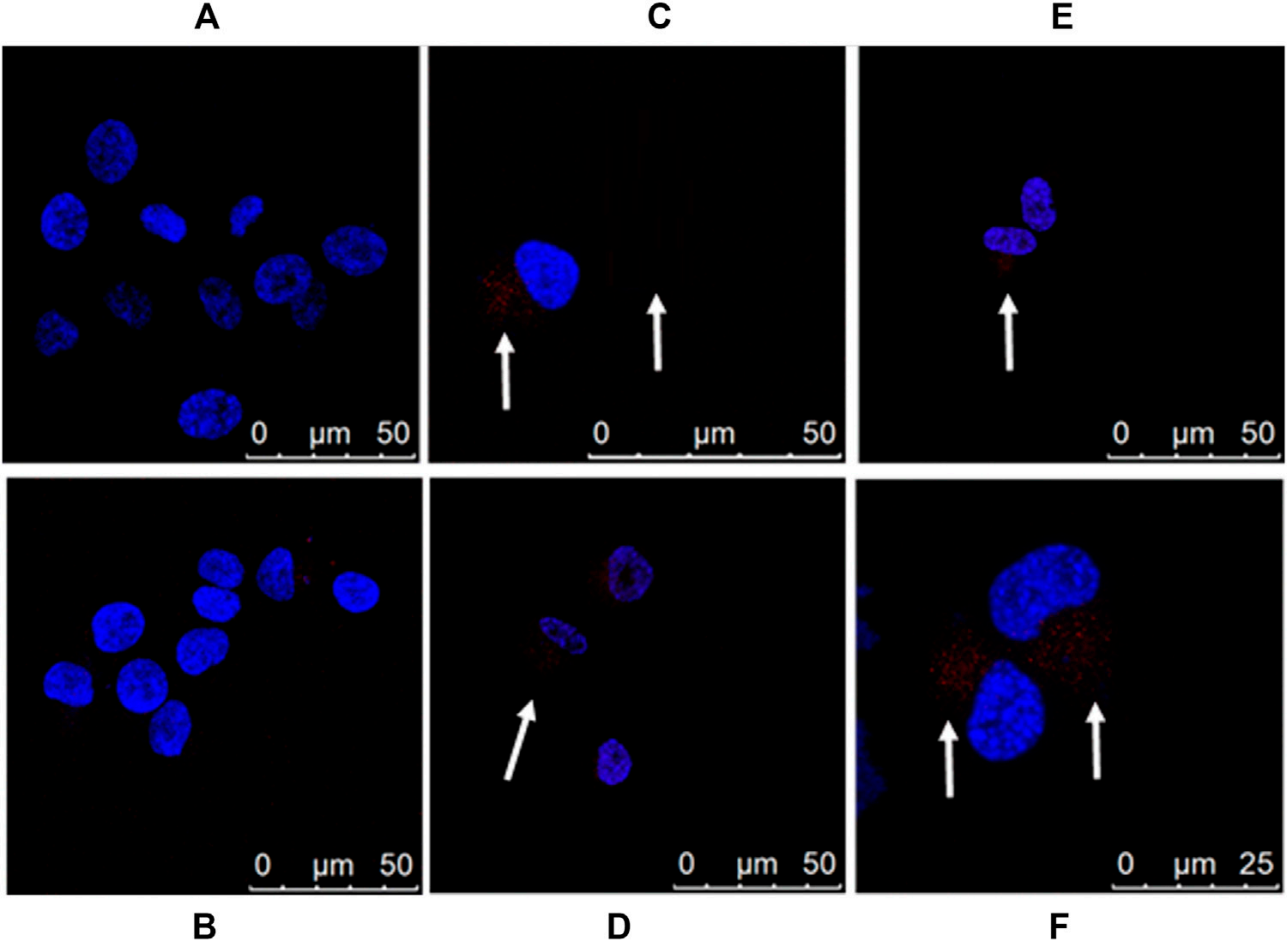
Imaging of U87MG cells by APTSAP-protoporphyrin IX. U87MG cells were incubated for 2 h with 5 µM APTSAP-PPIX complexes and 5 µM PPIX and observed under a confocal microscope. **(A)** control; **(B)** PPIX; **(C–F)** APTSAP-PPIX.

We also examined the APTSAP-PPIX-nucleolin interaction by confocal microscopy (Figure 10). As shown, there is evidence of the accumulation of a fluorescence signal in a perinuclear zone (Figure 10, see arrows), strongly suggesting transport of APTSAP inside the target cell.

### Reverse Transcription Polymerase Chain Reaction Experiments

To verify the transcription of the saporin gene, as a further confirmation of the intracellular delivery of APTSAP, total RNA was extracted from U87 and 3T3 cells after exposure to 20 nM APTSAP for 96 h, and then a RT-PCR experiment was carried out (see *Materials and Methods*).

As a positive control, we used a pET11-d plasmid carrying the wild type saporin gene (pET11d-SAP).

**FIGURE 11:**
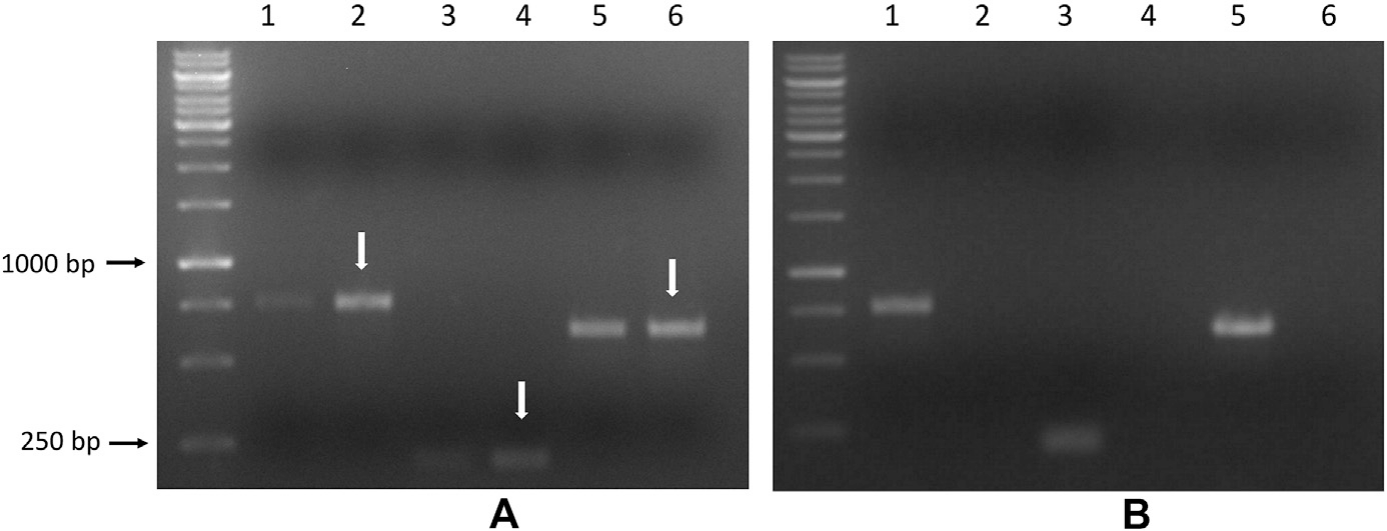
U87 **(A)** and 3T3 cells **(B)** were treated with 20 nM APTSAP for 96 h, total RNA was extracted from cell pellets. PCR was performed on the corresponding cDNA using the three couples of primers, as described in the *Materials and Methods* section. pET11d plasmid carrying the wild type saporin gene (pET11d-SAP) was used as a positive control. Amplified products are indicated by arrows, and the expected dimensions are: FW1/RV1:765 bp, FW2/RV2: 204 bp, FW3/RV3: 621bp. Lanes 1-3-5: pET11d-SAP; lanes 2-4-6: treated.

As shown in Figure 11 and in Supplementary Figures S6, S7, distinct fragments from the saporin transcript product were generated by RT-PCR qualitative analysis from 1 μg of total RNA extracted from U87 cell pellets but not from the 3T3 cells after 96 h of APTSAP treatment.

The observed cytotoxic activity on U87MG cells seems to correlate with the expression of the saporin gene within the tumor cells.

## Discussion

Glioblastoma multiforme represents an incurable disease, being refractory to most common anticancer therapies (Batash et al., 2017). To date, only an anti-angiogenic agent, bevacizumab has been recently approved for the treatment of recurrent GBM in the United States and Canada. However, GBM has still a poor prognosis showing a high rate of recurrence, following surgery and chemotherapy (Polivka et al., 2017; Bahadur et al., 2019). Currently, temozolomide (TMZ) is the first choice for the treatment of GBM patients despite median overall survival is no longer than 15 months. Thus there is an urgent need for new therapies to treat this disease. In this perspective, the combined use of gene therapy approaches might be very attractive.

Indeed, the delivery of genes encoding toxic molecules into cancer cells has been successfully explored since decades (Fitzgerald and Pastan, 1989; Kawakami et al., 2006) including the use of toxin genes (Castro et al., 2011) such as PE (Pseudomonas exotoxin A).

Among toxins possibly useful to target cancer cells, saporin RIP is a potent protein synthesis inhibitor and should be considered (Polito et al., 2013).

In this paper, we describe the non-viral delivery of the saporin gene to glioblastoma cells which is mediated by an aptamer, targeting glycosylated nucleolin, a cell surface marker of gliomas (Hovanessian et al., 2010). Our previous data demonstrated that nucleolin expression at the cell surface increases with the GBM malignancy grade, thus indicating that it constitutes a useful histopathological marker for glioblastoma grading, as well as, for targeted therapy approaches (Galzio et al., 2012; Dhez et al., 2018).

Recently, the aptamer AS1411 has been used to target cancer cells overexpressing glycosylated nucleolin on their cell surface and has entered clinical phases (Laber et al., 2007; Rizzieri et al., 2010; Koutsioumpa and Papadimitriou, 2014). The clinical efficacy of naked AS1411 has proven to be limited by a reduced persistence of the synthetic DNA in the bloodstream, and therefore more recently AS1411 oligonucleotide has recently been incorporated (as a component) in nanocomposite materials (nanoparticles) to exploit its specific binding capacity and selectivity towards nucleolin, for the delivery of anticancer drugs (Ghahremani et al., 2018; Zhao et al., 2019).

As described previously in different studies (Bates et al., 2009; Soundrajan et al., 2009), several tumor cell lines were assayed to evaluate the optimal biological concentration of AS1411, and in all the cases examined, 50% of cytotoxicity resulted in a concentration range between 2 and 10 µM to achieve the latter concentrations, cells needed a continuous AS1411 exposure in order to elicit the desired effect.

It has been demonstrated that the biological activity of aptamers is strictly associated with their ability to form G-quartets-containing structures (Bates et al., 1999) that can bind nucleolin, thus leading to cell arrest in the S-phase, after several cell cycles (Xu et al., 2001).

However, it is also known that AS1411 does not cause a rapid cytotoxic effect when added to cells. In contrast, cytostasis depends on the inhibition of cell division, and for the cell death induction a prolonged exposure is needed (4–6 days) (Bates et al., 2009).

Aptamers, unlike antibodies, are not associated with serious side-effects in infused patients because they are essentially not immunogenic (Esposito et al., 2011), thus representing a suitable delivery system for drugs or toxins to target cancer cells. Furthermore, aptamers have also been incorporated in the terminal loop of shRNAs (Pang et al., 2018), thus allowing expression of these constructs in HIV-infected cells targeting the viral integrase protein.

AS1411 has been conjugated with different types of drugs, nanoconjugates, toxins, but no evidence, to the best of our knowledge, is presented in literature of the use of this aptamer as a double-stranded DNA, as we did for the first time here. Our results show that AS1411 double stranded DNA at the 5′ of APTSAP plasmid forms G-quadruplexes, as well and it is capable of efficiently binding cell surface nucleolin in U87 cells.

After nucleolin-binding, the saporin gene can be delivered inside the cell, for transient expression and saporin exerts its RIP biological activity.

In our experiments, 50% reduction of U87 viability was reached using an average 24–30 μg/ml saporin gene concentration, showing a selective targeting of glioblastoma U87 cells: no such toxic effect could be observed in 3T3 control cells. In addition, the saporin N-glycosidase activity and cytotoxicity on U87 cells were directly caused by the RIP activity, since we could observe that the KQ saporin catalytic inactive mutant did not show any cytotoxic effect.

Overall our data suggest that plasmids like APTSAP incorporating a double stranded aptamer DNA could be a promising delivery system to vehiculate saporin genes into target cancer cells, even much more efficiently when complexed to polycationic carriers, such as PEI. These polycationic carriers, were proposed by Behr to undergo the “proton sponge” hypothesis such that their efficiency relies on extensive endosome swelling and rupture that provides an escape mechanism for the polycation/DNA particles from the endo/lysosomal compartment (Behr, 1997) as one of the most generally accepted intracellular delivery mechanism, although recently heavily debated concerning one (mis)interpretation that lysosomal pH is modified (Benjaminsen et al., 2013). In an elegant study using real time microscopy Zuhorn’s lab members (Rehman et al., 2013) confirmed that the “proton sponge mechanism” may play a major role in release of nucleic acids from PEI-polyplexes from endosomes, although they did not observe a complete endosome lysis but instead a “bursting” of the endosomal membrane with only one up to four/five nanocarriers per cell, releasing nucleic acids from the acidic endosomes. This observation supports the suicide gene delivery approach described here, and thus, PEI-polyplexes might not represent a bottleneck.

PEI has a high concentration of positively charged nitrogen atoms, which makes it suitable for condensing large negatively charged molecules such as plasmid DNA, resulting in the formation of polyplexes. Endocytosis may be, indeed, the main entry route of PEI Polyplexes that have been co-localized with LAMP-1 in the lysosomes (Benjaminsen et al., 2013).

Cytotoxic saporin activity was in fact early revealed after one day when GBM cells were exposed to APTSAP/PEI polyplexes. PEI was in fact able to shorten the toxicity onset in U87 cells when compared to the naked APTSAP DNA. 3T3 cells viability was on the contrary not affected at 24 h and only slightly at 48 h, as a possible minimal non specific delivery of saporin gene, if any.

Futhermore, we have also shown here that APT-SAP-mediated cell death of U87 cells is not due to apoptosis but rather could be explained by autophagy mechanism(s) or by a new cell death pathway, recently identified, called methuosis, firstly characterized in cancer cells, such as MDA-MB-231 breast cells showing enlarged nuclei and cytoplasm (Reyes-Reyes et al., 2015).

Cell death mediated by autophagy is one of the most widely studied non-apoptotic cell death mechanisms which has been reported in several types of cancer cells (Bursch et al., 2000), including glioblastoma, for instance after treatment with temozolomide (Würstle et al., 2017), 4-Nerolidylcatechol (Massaro et al., 2017), or with ionizing radiation (Yao et al., 2003) and the anti-angiogenic compound bevacizumab (Castro and Aghi, 2014); however it is not yet clear whether these increased autophagic activities are the primary cause for cell death.

An alternative view suggests that the autophagic activity is a survival cell response to stress conditions (Levine and Yuan, 2005). Methuosis and methuosis-like vacuolization events can be induced by treatment with different types of chemical compounds both in cultured cells and tissues, such as tertiary and organic amines (Yang et al., 1965; Aki et al., 2012), chemotherapy drug F14512 (Brel et al., 2011), and indole-based chalcones (Robinson et al., 2012).

Recently, methuosis has been proposed as the main cell death mechanism induced by AS1411 signaling (Esposito et al., 2011). AS1411 binding to nucleolin leads to sustained activation of Rac1 and causes a hyperstimulation of macropinocytosis (Reyes-Reyes et al., 2015). Clearly, the morphological features we observed are distinct from those seen in cells undergoing apoptosis: apparently there is no chromatin condensation nor DNA fragmentation which are typical signs of apoptosis mechanism(s), whereas the cells are filled with enlarged vacuoles and membranes debris, as during necrosis-related events.

In conclusion, we show here that uncomplexed APTSAP plasmid DNA didn’t show any specific toxicity on 3T3 cells: we suggest it could be also effective if used in a local treatment protocol of GBM animal model, as well as in its polyplexed form, APTSAP/PEI. Once bound to the exposed nucleolin, APTSAP (presumably by exploiting its G4 structure) following nucleolin-mediated internalization and endosomal escape could support the saporin gene transient expression causing cell intoxication. Polyplexed DNA complexes with PEI generally show an increased stability in human serum (Moret et al., 2001; García et al., 2012) thus supporting the use of this complexed form of DNA for GBM treatment. In this kind of tumor, the BBB is generally damaged, thus allowing for delivery of drugs *via* systemic injection, however, due to the particular CNS location, it would be possible and may be preferred, to treat the patients *via* local infusions or even allowing the release of drugs by automated and programmable devices (Kaurav and Kapoor 2017; Yu et al., 2019).

Overall our main findings support the proof-of-concept of using APTSAP PEI-polyplexes for local delivery in rat glioblastoma model with an intrathecal delivery (Oh et al., 2011) and on the basis of these encouraging results, we plan to perform future experiments on *in vivo* tumor models.

## Data Availability

The raw data supporting the conclusions of this article will be made available by the authors, without undue reservation.
