# Supplementary figures and images for: Aptamer-Driven Toxin Gene Delivery in U87 Model Glioblastoma Cells
Source: Front Pharmacol. 2021 Apr 15;12:588306. doi: 10.3389/fphar.2021.588306 (PMC8082512; doi:10.3389/fphar.2021.588306)

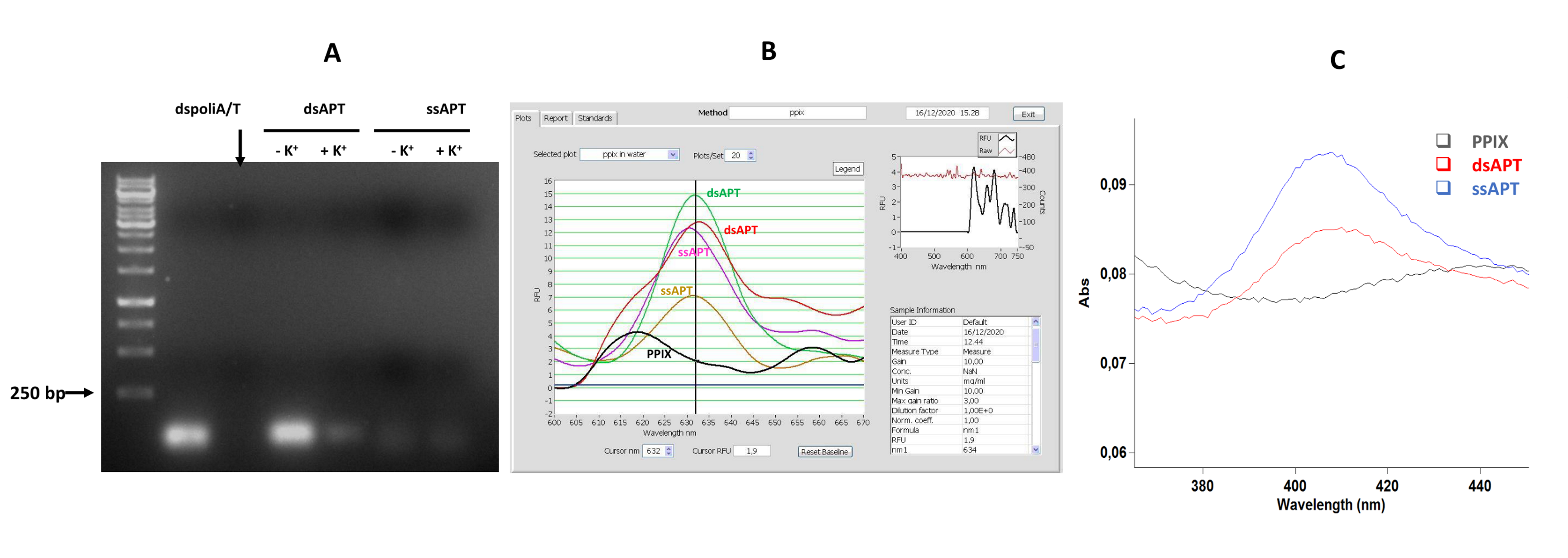

Supplement: Supplementary file 1 [file image1.tiff]

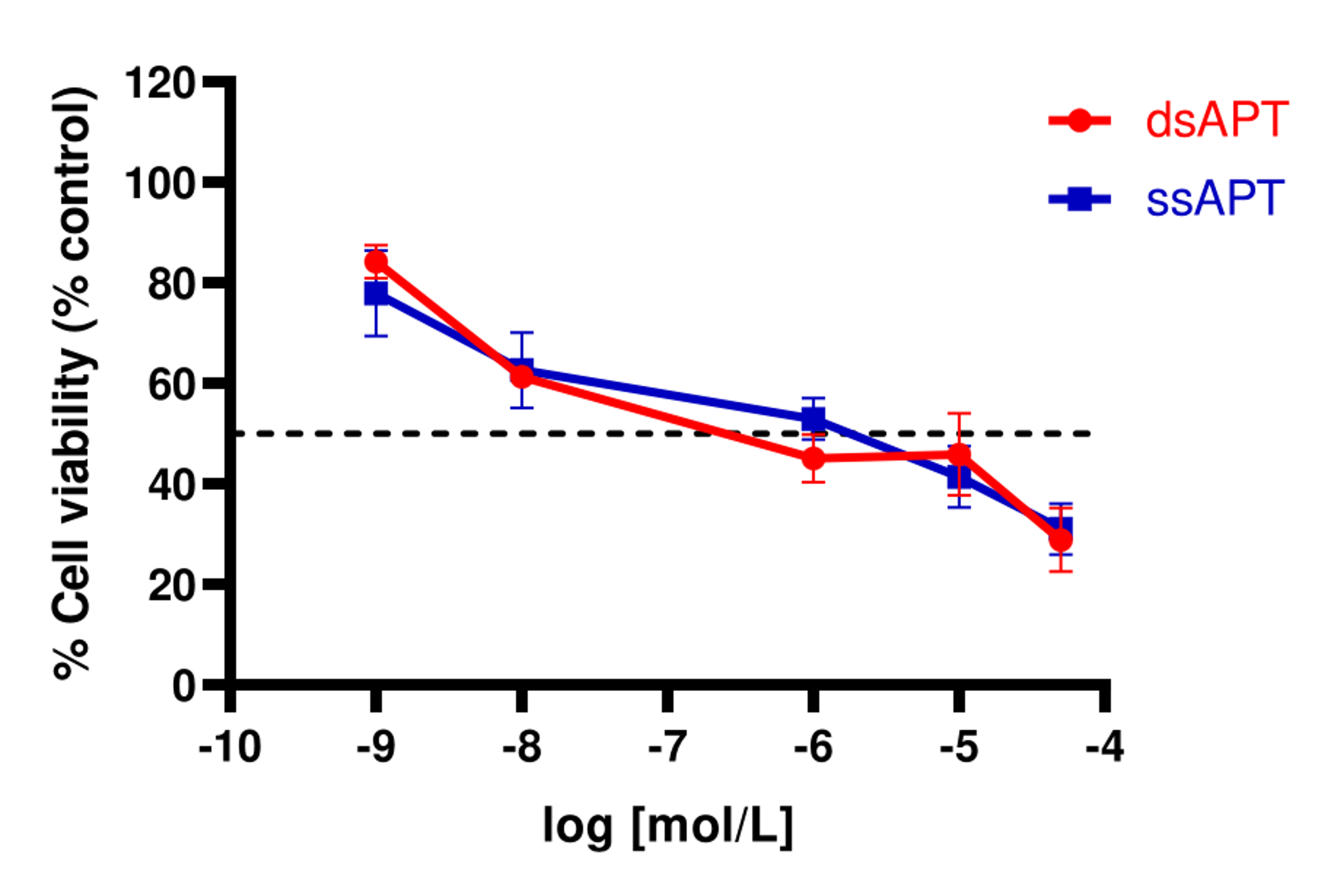

Supplement: Supplementary file 2 [file image2.tiff]
